# Cohort Profile: VZNKUL–NMIBC Quality Indicators Program: A Flemish Prospective Cohort to Evaluate the Quality Indicators in the Treatment of Non-Muscle-Invasive Bladder Cancer

**DOI:** 10.3390/cancers16213653

**Published:** 2024-10-29

**Authors:** Murat Akand, Ralf Veys, Dieter Ost, Kathy Vander Eeckt, Frederic Baekelandt, Raf Van Reusel, Pieter Mattelaer, Loic Baekelandt, Ben Van Cleynenbreugel, Steven Joniau, Frank Van der Aa

**Affiliations:** 1 Department of Urology, University Hospitals Leuven, 3000 Leuven, Belgium; murat.akand@uzleuven.be (M.A.); loic.baekelandt@uzleuven.be (L.B.); ben.vancleynenbreugel@uzleuven.be (B.V.C.); steven.joniau@uzleuven.be (S.J.); 2 Laboratory of Experimental Urology, Urogenital, Abdominal and Plastic Surgery, Department of Development and Regeneration, KU Leuven, 3000 Leuven, Belgium; 3 Department of Urology, AZ Groeninge, 8500 Kortrijk, Belgium; ralf.veys@azgroeninge.be; 4 Department of Urology, AZ Sint Blasius, 9200 Dendermonde, Belgium; dieter.ost@azsintblasius.be (D.O.); kathy.vandereeckt@azsintblasius.be (K.V.E.); 5 Department of Urology, AZ Sint-Lucas Brugge, 8310 Brugge, Belgium; f.baekelandt@telenet.be; 6 Department of Urology, AZ Turnhout, 2300 Turnhout, Belgium; raf.vanreusel@azturnhout.be; 7 Department of Urology, AZ Oostende Damiaan, 8400 Oostende, Belgium; pieter.mattelaer@azoostende.be

**Keywords:** bladder cancer, non-muscle-invasive, registry, quality indicator, recurrence, progression, prospective, survival, treatment

## Abstract

**Purpose:** Bladder cancer (BC) is a heterogeneous disease with varying outcomes, influenced by disease heterogeneity and variability in treatment and follow-up. Risk groups have been established for non–muscle-invasive BC (NMIBC) to standardize therapy, and several quality control indicators (QCIs) monitor adherence to these risk group-based guidelines. However, controversial results had been obtained regarding the oncological benefits of these QCIs until recent high-quality studies from large registries showed their usefulness. To improve adherence to the European Association of Urology (EAU) Guidelines and benchmark current care in Flemish hospitals within Vlaams Ziekenhuisnetwerk–KU Leuven (VZNKUL), a QCI program for NMIBC was initiated in 2013. This study aims to describe the demographic, clinical, and treatment data of patients enrolled in this program. **Participants:** The VZNKUL–NMIBC Quality Indicators Program Registry is a prospective cohort including patients treated and followed up with at seven academic and non-academic Flemish hospitals since June 2013. Data collection includes patient characteristics, tumor data, treatment, and oncological outcomes. **Findings to date:** From June 2013 to December 2020, 4744 transurethral resections of bladder tumors (TURBTs) from 2237 unique patients were analyzed. Most patients (80%) were men with a median age of 73. The median time from diagnosis to TURBT was 19 days. A single tumor was detected in 37% of TURBTs. Tumors larger than 3 cm were found in 20.8% of cases. In 46% of TURBTs, a reTURBT was scheduled according to guidelines. The complication rates were 7.5% and 2.4% for bladder perforation and bleeding, respectively. Postoperative single intravesical instillation of chemotherapy (SIVIC) was administered to 56.9% of 1533 indicated patients with a median time to administration of 4.7 h. Among the cohort, 60.4% had NMIBC, and 9.3% had muscle-invasive BC. Of 972 high-risk patients, 60.7% received adequate BCG induction, while 39.4% received adequate maintenance. After BCG induction ± maintenance, 39.7% were tumor-free, with 17.7% recurrence and 4% progression to muscle-invasive BC. BCG treatment was terminated early for 17% of patients due to intolerance. Early cystectomy was performed for 2.4% of the BCG-naïve patients, and 27.7% of patients with BCG failure underwent a BCG rechallenge. For intermediate-risk patients, 2.1% received adequate BCG, and 23% received intravesical chemotherapy. The median follow-up was 57 months. Five-year recurrence-free, progression-free, cancer-free, overall, and cancer-specific survival rates were 53%, 91.6%, 89%, 70.6%, and 95.6%, respectively, for the NMIBC patients. Of 400 non-metastatic MIBC patients, 217 (54.3%) underwent radical cystectomy (RC), of whom 46% received neoadjuvant chemotherapy, while 18 (4.5%) refused RC, and 74 (18.5%) were considered unfit for the surgery. **Future plans:** The VZNKUL–NMIBC Quality Indicators Program Registry will continue collecting data to evaluate QCIs and monitor treatment quality, enabling hospitals to benchmark their performance and improve patient care. Additionally, the registry’s real-world data can support research and international collaboration. **Trial registration:** The study was registered on ClinicalTrials.gov (NCT04167332).

## Simple Summary

Bladder cancer is the ninth most commonly diagnosed form of cancer in both sexes worldwide and the fifth in Europe. The most common form, non-muscle-invasive bladder cancer, is highly prevalent with high recurrence rates and has wide range of outcomes that is partially due to the variability in the treatment delivered. Therefore, some recommendations have been done by the European Association of Urology to standardize their management. Several quality control indicators have been proposed to monitor the adherence of urologists and hospitals to these recommendations. A quality control indicator program has been initiated in the hospitals that are a part of the Flemish Hospital Network in June 2013 using a specific prospective registry. We have recently published the first analysis of these quality control indicators, which showed significant differences between the hospitals. Before publishing the second analysis with more patients and additional quality control indicators, we aimed to describe the characteristics of the cohort in this study.

## 1. Introduction

Bladder cancer (BC) is the ninth most commonly diagnosed form of cancer in both sexes worldwide and the fifth in Europe [1,2]. In Belgium, 2364 new cases of BC were diagnosed in 2013, which is expected to rise to 2900 by 2025 [3]. A similar anticipation has been expressed by the World Health Organization (WHO), suggesting that the number of BC cases and deaths would almost double in the near future, parallel to the increase in life expectancy [4]. Although BC is more commonly seen in (predominantly white) males, females, and Black individuals are more frequently diagnosed with advanced-stage disease [5,6]. Most BC cases are urothelial carcinoma (UC) in subtype, and approximately 75% of these are non–muscle-invasive BC (NMIBC). The disease is highly prevalent due to its indolent natural history and high recurrence rate [7]. Most BC cases are associated with external risk factors, of which approximately 50% are caused by tobacco smoking. At the same time, occupational carcinogen exposure is a significant risk factor for a subset of patients, especially in industrialized countries [8].

Even though our insight into this disease’s molecular mechanism(s) has increased significantly in the last decade, much is still unknown about this complex disease. Its wide range of outcomes is attributed to the intrinsic disease heterogeneity and the variability in delivered treatment and follow-up. To standardize therapy based on the risk of disease recurrence and/or progression, several prognostic models have been developed. The European Association of Urology (EAU) has proposed categorizing patients into four risk groups: low-, intermediate-, high-, and very high-risk [9,10,11,12]. Utilizing this prognostic risk stratification, the EAU Guidelines Office has published its recommendations for the diagnosis, treatment, and follow-up of NMIBC patients [7]. Despite these guidelines, it has been consistently observed, both in Europe and globally, that adherence to the recommended guidelines is suboptimal [13,14,15,16,17,18]. Furthermore, the efficacy of these guidelines in improving oncological outcomes has not been widely demonstrated. Some retrospective studies have reported that adherence to guideline-recommended practices, such as second transurethral resection of the bladder tumor (TURBT), adjuvant bacillus Calmette–Guérin (BCG) maintenance treatment, and regular cystoscopic surveillance, significantly impacts recurrence-free survival (RFS) and recurrence/progression rates [15,19,20]. Recently, Mariappan et al. published results from the Scottish Bladder Cancer Quality Performance Indicators Influencing Outcomes, Prognosis and Surveillance (Scot BC Quality OPS) project, which demonstrated for the first time that achieving predefined quality control indicator (QCI) targets in benchmarking centers reduced recurrence and progression rates in NMIBC [21].

A QCI program specific to NMIBC was initiated at three Flemish hospitals, with a specific registry established in June 2013, aiming to increase adherence to the EAU Guidelines and to benchmark the current standard of care at the hospitals affiliated with Vlaams Ziekenhuisnetwerk–KU Leuven (VZNKUL; Flemish Hospital Network) [22]. Since then, patients undergoing TURBT have been prospectively registered in this database across seven participating hospitals with, on average, more than 700 TURBTS for more than 600 unique patients per year during the last five years. Apart from increasing adherence to the EAU Guidelines and benchmarking the participating hospitals, this QCI program also aims to find answers to several research questions, such as those concerning the effect of guideline adherence on recurrence/progression rates and survival outcomes and reasons for non-compliance. The first analysis of six QCIs (complete resection [CR] status, presence of detrusor muscle [DM] in the specimen, postoperative single intravesical instillation of chemotherapy [SIVIC], repeat TURBT [reTURBT], start of BCG induction therapy, and performing TURBT within six weeks after diagnosis) from the first three participating hospitals was recently published. This analysis revealed that the quality of reporting and compliance with these QCIs were suboptimal, with significant variability between centers. Moreover, postoperative SIVIC correlated with RFS [23].

In this article, we describe the VZNKUL–NMIBC Quality Indicators Program cohort and report on patient and tumor characteristics at enrollment, surgical treatment information, adjuvant therapy patterns, follow-up, and survival information prior to publishing the second analysis on QCIs. This study will reveal comprehensive insights into patient and treatment characteristics with real-world data before analyzing the guidelines adherence and their impact on disease outcomes.

## 2. Cohort Description

This prospective registry started at one academic and two non-academic hospitals in Flanders, utilizing a specifically developed electronic case report form (eCRF) for TURBT. This eCRF was embedded in the electronic patient file system known as Klinisch Werkstation (KWS). The registry protocol was approved by the institutional review board (Clinical Trials Center UZ Leuven) in June 2013, and it was approved by the Ethics Committee Research UZ/KU Leuven after several amendments (approval number: S55725, approval date: 6 June 2014). Written informed consent was obtained from every included patient. This registry has been registered at ClinicalTrials.gov (NCT03973671), and the details of its development have been previously published elsewhere [22]. To encompass more aspects of the management of NMIBC patients, three additional eCRFs have been implemented (for bladder instillations, follow-up, and multidisciplinary team meetings [MDT]). The number of participating hospitals has increased to seven. The selection of the centers was voluntary, and it depended not on the volume of TURBTs they performed but on their willingness to participate and use the eCRFs. This approach ensured coverage across both academic and non-academic, as well as low- and high-volume, centers.

The numbers of TURBTs, bladder instillations, follow-ups, MDTs, and unique patients since the inception of each form are listed in Supplementary Tables S1–S4, categorized per year and center. These data demonstrate an increase in the number of registered procedures and patients over time, attributable to the growing number of participating centers and improved compliance with using the eCRFs. Aiming to increase the quality of care and provide a benchmark for participating centers, this program will continue indefinitely, contingent on the voluntary participation of the centers. Here, we report on patients registered up to the end of 2020 in order to achieve concordance with the upcoming new analyses on QCIs. Additionally, reTURBTs for these patients up to the point of data extraction have been included to provide more insight into their longitudinal disease trajectory.

### 2.1. Demographic and Medical History Information

While reporting on the cohort, we grouped the NMIBC patients according to the risk stratification outlined in the EAU 2019 Guidelines (low-, intermediate-, and high-risk). At some points, the highest-risk subgroup within the high-risk group was specifically highlighted [24]. Patients diagnosed with muscle-invasive bladder cancer (MIBC) were presented as a separate group. Patients’ ages were recorded at the time of the first registered TURBT. The American Society of Anesthesiologists (ASA) score on physical status was manually retrieved from the anesthesiology report of the first registered TURBT (from available patients). The Charlson comorbidity index (CCI) at the time of the first registered TURBT was either retrieved from the MDT form (for the centers using this eCRF) or manually calculated by thoroughly evaluating patients’ electronic medical records. Smoking status and history of pelvic radiotherapy were manually extracted from patients’ electronic medical records. Patients who had ceased smoking at least six months before the first registered TURBT were categorized as former smokers.

### 2.2. Surgical and Adjuvant Treatment Information

Macroscopic tumor characteristics and surgical data were retrieved automatically from the surgery form, which all centers used. Pathological data were retrieved either from the MDT form (for the centers using this eCRF) or manually from the pathology reports of each TURBT. Tumor stage and grade were reported using the American Joint Committee on Cancer (AJCC) Tumor-Node-Metastasis (TNM) staging (8th edition) and the WHO classification (1973 and/or 2004/2016), respectively. Data on adjuvant intravesical treatments (BCG and chemotherapy) were either retrieved from the instillation forms (for the centers using this eCRF) or manually from patients’ follow-up files. Adequate BCG exposure was defined according to the Food and Drug Administration (FDA) criteria, which specify at least five out of six instillations of the induction course plus at least two out of three instillations in the first cycle of maintenance therapy or at least five out of six instillations of initial induction course plus at least two out of six instillations of the second induction course [25]. The categories of BCG failure were defined according to the EAU Guidelines as BCG-refractory tumor, BCG-relapsing tumor, BCG-unresponsive tumor, BCG intolerance, and MIBC [24]. For patients with recurrent high-risk tumors, we pragmatically established inclusion/exclusion criteria to decide whether that recurrence could be used for the BCG-related analyses. Patients who had received at least adequate BCG treatment after a high-risk tumor episode and remained tumor-free for at least two years from the completion or discontinuation of BCG treatment till recurrence were considered new cases for the analysis. Conversely, patients who did not receive or received inadequate BCG instillations were re-included in the analysis if they experienced a high-risk tumor recurrence warranting BCG treatment at least one year after the discontinuation of BCG or the detection of their previous tumor. We also adopted a pragmatic stratification of disease outcomes for non-BCG-treated patients to reflect patient outcomes better. This included the following categories: no recurrence, low-grade (LG) recurrence (TaLG tumors), high-grade (HG) recurrence (TaHG and Tis tumors), T1 tumors (representing tumor upstaging for TaLG, TaHG, and Tis), muscle-invasive disease, and metastatic disease. All other data parameters reported here were automatically retrieved from the registry’s eCRFs.

### 2.3. Follow-Up and Survival Information

Recurrence was defined as the reappearance of a tumor of any T-stage and grade, while progression was described as a tumor with a T-stage of at least T2 or metastatic disease. Patients are being followed up with according to the most recent EAU Guidelines recommendations relevant to their risk groups. The time between the dates of TURBT and recurrence at the level of surgical intervention was defined as RFS. Cases without recurrence were censored at the time of the next TURBT (reTURBT) or the last follow-up. The time between the date of the first TURBT and the dates of progression, cystectomy, and death from any cause were calculated as progression-free survival (PFS), cystectomy-free survival (CFS), and overall survival (OS), respectively. These were defined at the patient level. Cases without the respective events were censored at the last follow-up. Cancer-specific survival (CSS) was the time between the date of the first TURBT and the date of death due to BC, also defined at the patient level. Death from other causes was treated as a competing event, and patients who were alive were censored at the last follow-up.

### 2.4. Statistical Analysis

Kaplan–Meier estimates were used to construct PFS, CFS, and OS curves. The cumulative incidence function was used for CSS. Survival curves for RFS were derived from a Cox model using the robust sandwich estimate of Lin and Wei to account for the clustering of interventions among patients [26]. Continuous variables were summarized as the median and interquartile range (IQR), and categorical variables were shown as the frequency count (*n*) and percentage. Analyses were performed using SAS software (SAS System for Windows, version 9.4, SAS Institute Inc., Cary, NC, USA).

## 3. Findings to Date

### 3.1. Patient Characteristics

**Figure 1:**
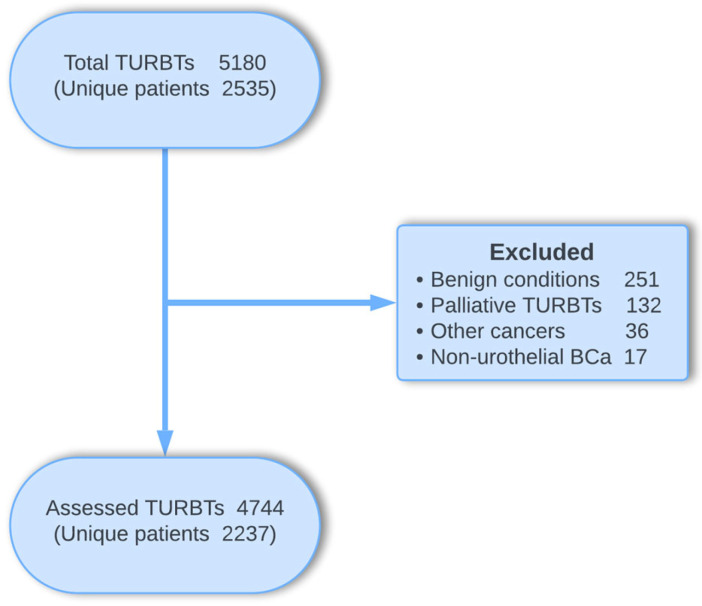
Flowchart of the included and excluded TURBTs/patients.

After excluding surgeries performed for palliative purposes (for patients with metastatic disease or those unable to undergo radical cystectomy [RC]), bladder tumors other than UC, other pelvic cancers, and benign conditions, a total of 4744 TURBTs for 2237 unique patients were analyzed (Figure 1 and Supplementary Table S5). Approximately 80% of the patients were men, and 70% were either active or former smokers. The median age at the first registration was 73 years (range: 29–101), with 62.7% of the patients older than 70 years. All patient characteristics are presented in Table 1.

### 3.2. Surgical Characteristics

Among the analyzed TURBTs, 1702 were performed as the first TURBT for a primary tumor, while 528 were reTURBT for patients registered for the first time. Together, for primary and recurrent tumors, the median time from diagnosis to TURBT was 19 days, and the median operation duration was 20 min. Remarkably, visual enhancement techniques (such as blue light fluorescence or narrow-band imaging) were used in only approximately 10% of the operations. Of the 1146 indicated TURBTs, reTURBT was planned for 527 (46%) cases, of which 91.2% could be performed. The complication rates of 7.5% and 2.4% for bladder perforation and bleeding, respectively, were consistent with the literature data. Out of 1533 indicated patients, postoperative SIVIC was requested for 62.4%, of whom 56.9% received it. The median time from request to receipt was 282 min (4.7 h; IQR: 3.2–7.8 h). Other peri-operative characteristics are presented in Table 2. The reasons for not requesting postoperative SIVIC in 32.6% of the indicated patients are listed in Table 3. A significant majority of the cases (86.9%) were discussed at an MDT.

### 3.3. Tumor Characteristics

The median number of tumors resected per TURBT was two, with 37% of the TURBTs having a single tumor and 20.8% exceeding 3 cm in their largest dimension. The tumors were mainly located on the lateral walls of the bladder (19.9% for both the left and right sides), while the involvement of the prostatic loge occurred only in 3.2% of cases. An NMIBC (Tis, Ta, and T1) was detected in 60.4% of the TURBTs, while MIBC was present in 9.3%. The WHO2004/2016 tumor grading system was more frequently used than the WHO1973 system, with 27.2% of tumors classified as HG tumors under the WHO 2004/2016 system. Both grading systems were applied to only 25% of 3311 indicated TURBTs. Ten (2.3%) muscle-invasive tumors and 59 (8.7%) T1 tumors were classified as either LG or grade 1/2. The distribution of the tumors according to the EAU risk stratification was 7.9%, 44%, 31.3%, and 16.8% for low-, intermediate-, high-, and highest-risk tumors. Patients were categorized in the same risk groups according to their first registered TURBT in 14.3%, 36.9%, 28.6%, and 20.3%, respectively. DM was sampled in 66.9% of the indicated TURBTs. Concomitant carcinoma in situ (CIS) and lymphovascular invasion (LVI) were reported in 10.3% and 2.8% of the tumors, respectively. Variant histology was detected in 9.7% of the tumors, with squamous, glandular, and micropapillary being the most common. Further details on tumor characteristics and the distribution of the variant types are listed in Table 4 and Supplementary Table S6, respectively.

### 3.4. Adjuvant Surgical and Intravesical Treatment Characteristics

**Figure 2:**
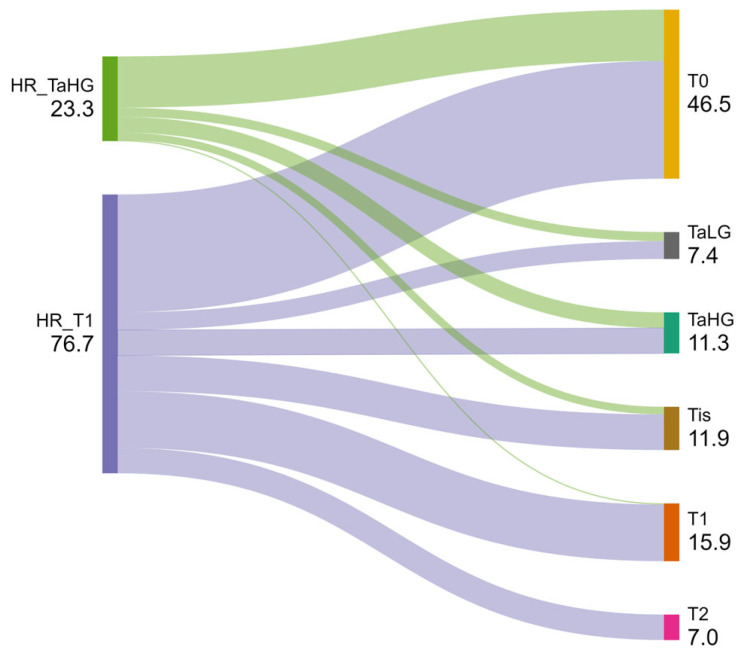
Distribution of the pathological results of the reTURBTs performed for TaHG (*n* = 76) and T1 (*n* = 251) tumors (made at SankeyMATIC.com). HG: high grade; HR: high-risk (to define the part of the tumor group for which reTURBT was performed); LG: low grade.

Of 76 TaHG tumors for which a reTURBT was performed, residual TaHG, TaLG, and Tis tumors were detected in 14 (18.4%), 8 (10.5%), and 7 (9.2%) cases, respectively. Additionally, 46 (60.5%) were tumor-free (T0), and 1 (1.3%) case was upstaged to T1 disease. Of 251 T1 tumors that underwent reTURBT, residual T1, TaHG, TaLG, and Tis tumors were detected in 51 (20.3%), 23 (9.2%), 16 (6.4%), and 32 (12.7%) specimens, respectively. Upstaging to T2 disease was observed in 23 (9.2%) cases, and 106 (42.2%) were tumor-free (T0) (Figure 2).

**Figure 3:**
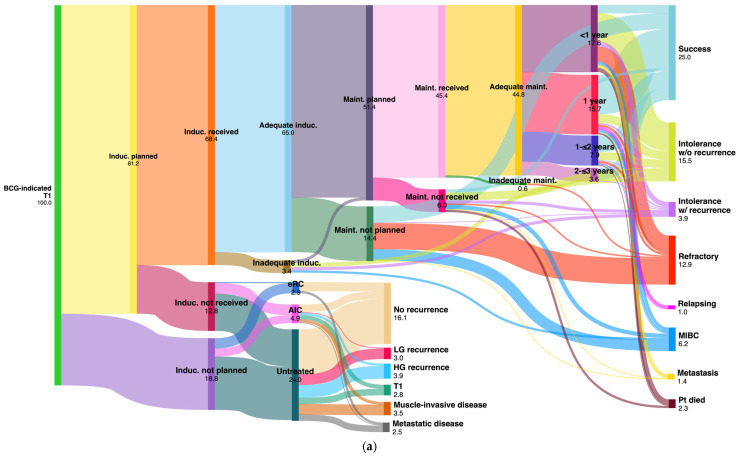

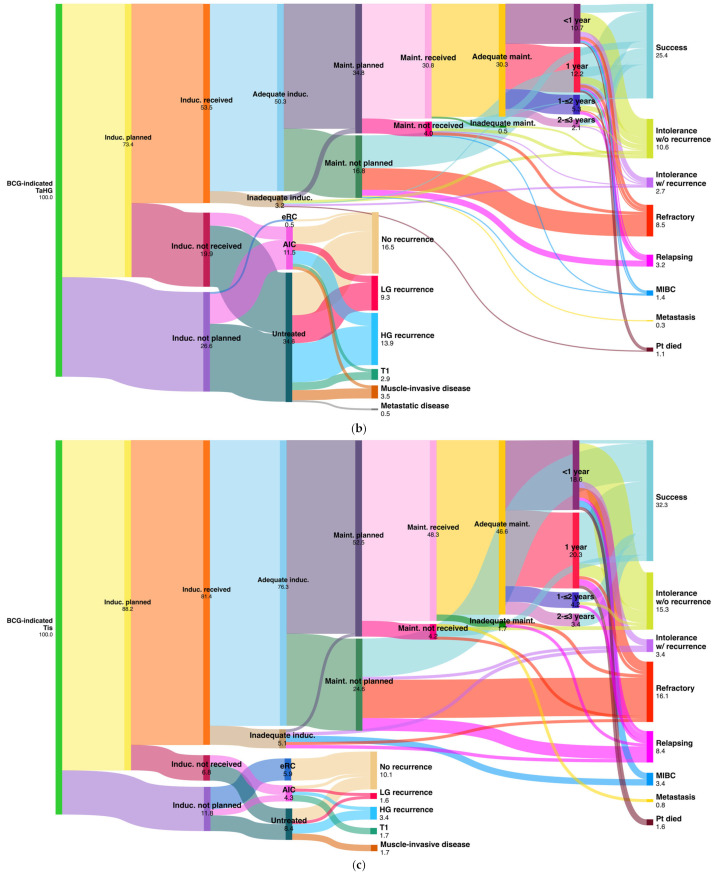

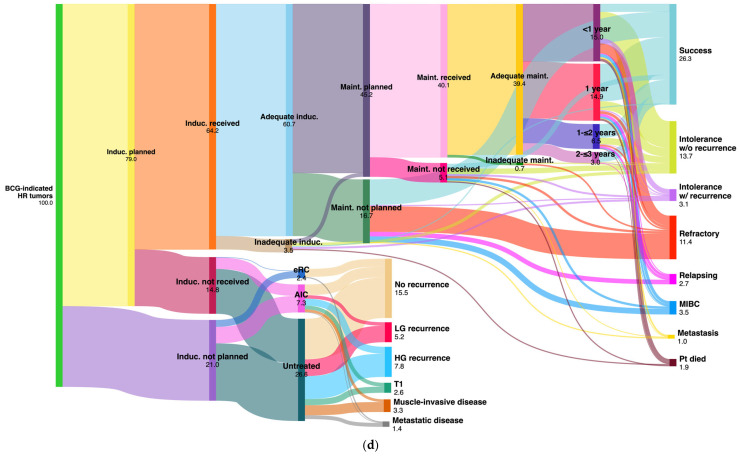
Sankey diagrams showing the proportions of the adjuvant BCG instillations and the results of BCG treatment for (**a**) BCG-indicated T1 tumors (*n* = 478), (**b**) BCG-indicated TaHG tumors (*n* = 376), (**c**) BCG-indicated Tis tumors (*n* = 118), and (**d**) BCG-indicated all HR tumors (*n* = 972) (made at SankeyMATIC.com). AIC: adjuvant intravesical chemotherapy; BCG: bacillus Calmette–Guérin; eRC: early radical cystectomy; HG: high grade; HR: high-risk; induc: induction; LG: low grade; maint: maintenance; MIBC: muscle-invasive bladder cancer; Pt: patient; w/: with; w/o: without.

Of 972 patients for whom high-risk BCG was indicated (Tis, TaHG, T1), BCG induction was planned for 79%, and 60.7% underwent adequate BCG induction. Maintenance therapy was planned for 45.2% of these patients, with 39.4% receiving adequate BCG maintenance. The distribution of patients receiving 1-, 2-, and 3-year maintenance therapy was 14.9%, 6.5%, and 3%, respectively. Only 7.4% of all BCG-indicated HR patients received intravesical chemotherapy (mainly Mitomycin C), primarily due to ineligibility for BCG due to intolerance or comorbidities and also influenced by recent BCG shortages. Detailed information about adjuvant intravesical treatments is presented in Table 5 and Figure 3.

After BCG induction with or without maintenance treatment (completed protocol or not), 26% of the patients achieved successful outcomes. This success rate increased to 39.7% when patients who experienced intolerance without recurrence were included, with higher rates observed in the Tis group (47.4%) compared to other risk groups. While 17.7% of the patients experienced recurrence (refractory or relapsing), progression to MIBC was observed in 4% of patients and metastatic disease in 0.9%. Meanwhile, intolerance led to an early termination of treatment for 17% of patients. The management of HR tumors and the results of intravesical BCG instillations for each group and in total are detailed in Table 6 and Figure 3. Early RC was performed for 2.4% of BCG-naïve tumors, with the highest rate seen in Tis tumors (5.9%), typically within a median time of 40 days from the decision taken at the MDT to perform surgery. Remarkably, intravesical chemotherapy was utilized more than twice as frequently for TaHG tumors compared to T1 tumors and nearly three times as frequently compared to Tis tumors.

Early RC (45%) and BCG rechallenge (26.5%) were the two most frequently chosen management options after BCG failure. However, out of 99 patients for whom RC was planned, 72 (72.7%) underwent surgery (with a median time of 48 days from MDT to surgery). The reasons for not proceeding with RC included patient refusal in 9 (9.1%) cases, medical unfitness in 16 (16.2%), and the detection of metastasis during staging or progression during neoadjuvant chemotherapy (NAC) in 2 (2%) cases. BCG rechallenge was particularly favored for Tis patients. Further details regarding the management of patients with BCG failure are summarized in Table 7.

Among the 1063 intermediate-risk patients, BCG induction was planned for only 3.9%, whereas only 2.1% received adequate BCG induction plus maintenance. Intravesical chemotherapy was administered to 245 patients (23%), of whom 226 (21.3%) received five or more instillations (range: 5–18). Only 14 (1.3%) completed 1-year maintenance. Detailed data regarding adjuvant intravesical treatments are provided in Table 5.

### 3.5. MIBC Patient Characteristics

Primary MIBC was detected in 322 (14.4%) patients, of whom 39 (12.1%) were metastatic at the time of diagnosis. Additionally, 117 patients (5.2%) were diagnosed with secondary MIBC. Of the 217 patients (54.3%) who underwent RC, 100 (46%) received NAC. Eighteen patients (4.5%) refused the operation, and 74 (18.5%) were deemed unfit for surgery. Thirty patients (7.5%) underwent bi-/trimodality treatment. The characteristics of the patients with MIBC are summarized in Table 8.

### 3.6. Follow-Up and Survival

**Figure 4:**
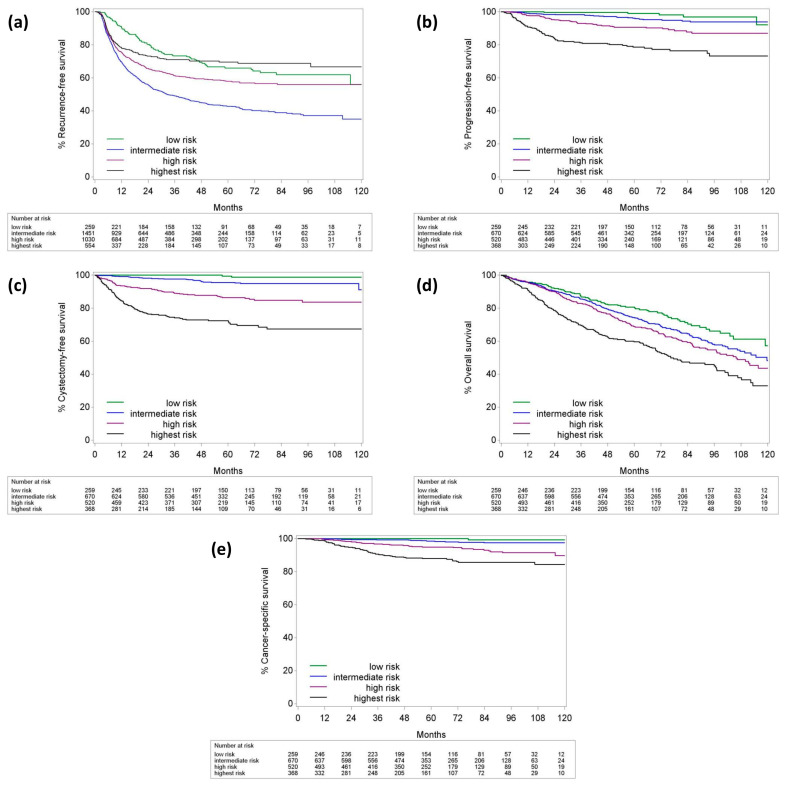
Survival curves of NMIBC patients according to risk groups. (**a**) Recurrence-free survival. (**b**) Progression-free survival. (**c**) Cystectomy-free survival. (**d**) Overall survival. (**e**) Cancer-specific survival.

During a median follow-up of 57 months (IQR: 35–83), recurrence was observed following 40.8% of the TURBTs. Among 1817 patients, 153 (8.4%) experienced disease progression, and 191 (10.5%) underwent RC. BC was the cause of death in 97 patients (5.3%), while 586 patients (32.3%) died from other causes. For NMIBC patients, the 5-year RFS, PFS, CFS, OS, and CSS estimates were 53%, 91.6%, 89%, 70.6%, and 95.6%, respectively. These figures, along with 2- and 10-year survival estimates for all patients and risk groups, are detailed in Table 9 and illustrated in Figure 4. During the same follow-up period, 37 patients (1.65%) developed a metachronous upper urinary tract urothelial carcinoma (UTUC), while 5 out of 125 patients (4%) with a prior UTUC history developed a metachronous tumor in their contralateral upper urinary tract.

## 4. Discussion

The VZNKUL–NMIBC Quality Indicators Program Registry is a prospective cohort of 4104 unique patients with BC (as of 1 May 2024) who were treated with TURBT plus adjuvant intravesical instillations (if indicated) and followed up with according to their pathological stage and risk stratification. Being the first of its kind in Belgium for BC, this registry offers a unique resource to gain insight into the quality of the care provided to NMIBC patients. It enables the benchmarking of participating centers regarding their adherence to QCIs, and it facilitates the provision of regular feedback to these centers. This feedback helps implement necessary improvements in QCIs for which suboptimal or below-threshold compliance has been detected. Additionally, the registry can serve as a tool for evaluating the outcomes of surgical and adjuvant treatments and their effects on oncological outcomes, as it provides invaluable real-world data representing different hospital settings.

In this cohort, the frequency of NMIBC, distribution of patients’ gender, and median age at diagnosis were consistent with the literature, as expected. The majority of patients underwent surgery within six weeks of diagnosis, which is a guideline-recommended threshold. Remarkably, both the WHO1973 and WHO2004/2016 tumor grading systems were used in only 25% of the reports. There are inconsistent results for using the three-tier (WHO1973) versus the two-tier (WHO2004/2016) tumor grading system. However, the most recent EAU Guidelines recommend using a hybrid grading system that subdivides grade 2 tumors into LG and HG categories [7]. Given that more research is needed to determine the most beneficial grading system, using both grading systems for each TURBT would be advisable.

ReTURBT was planned for only 46% of the 1146 indicated patients, while 42% actually underwent the procedure. While this rate appears low, it is difficult to draw definitive conclusions without an established threshold for this ratio. Although not systematically recorded in our registry, a review of patients’ files indicated that the main reasons for omitting reTURBT were surgeon or patient preference, often because of the patient’s age, general health status, or comorbidities. Given the ongoing debate about the added value of performing reTURBT, further research is needed to identify specific patient subgroups in which it could be safely omitted, thereby minimizing the economic and psychological burden of NMIBC treatment.

Postoperative SIVIC was requested for 62.4% of the patients by the operating urologists at the end of surgery, and 56.9% received the treatment. This falls below the arbitrarily identified threshold of 60% set by Mariappan et al. and the RESECT registry [21]. The primary reason for not administering SIVIC was the surgeons’ discretion, as some urologists believe it is not necessary or beneficial for some patients. Other significant reasons included bladder perforation (19%), very deep/extensive resection (13%), and the need for continuous irrigation (mainly due to bleeding—9%). These factors are directly related to performing an optimal and safe TURBT. It is obvious that, regardless of their experience level, urologists and residents should be better acquainted with tips and tricks for performing a complete and safe TURBT and understanding the oncological benefits of SIVIC. The median time of 4.7 h to receive postoperative SIVIC is consistent with the recommendation of 6 h of the EAU Guidelines. However, this result should be interpreted cautiously, as it was calculated from the data of only two centers, with the highest number of included patients.

Regarding adjuvant intravesical treatment, although BCG induction was planned for 79% of 972 BCG-indicated HR patients, only 60.7% received adequate BCG induction. Almost one-fifth of the patients for whom BCG induction was planned could not receive the treatment due to comorbidities, general status, age, intolerance, the BCG shortage, and the COVID-19 pandemic. The numbers of patients for whom maintenance was planned and those who received adequate BCG maintenance were even lower, at 45.2% and 40.1%, respectively. It is worth noting that some centers have used a four-cycle regime (at 3, 6, 9, and 12 months) for one-year BCG maintenance, while others have followed the three-cycle regime (at 3, 6, and 12 months), as recommended by the EAU Guidelines [7]. Our rates of postoperative SIVIC use and adjuvant BCG treatment were higher than those reported in the COBLAnCE cohort from France, which has been recently published [27]. As we have observed an increase in postoperative SIVIC use in our first analysis, we think creating the registry specifically for QCIs and the inherent Hawthorne effect might explain the difference in our cohort. Additionally, discussing the majority of the patients (86.9%) at MDT likely contributed to this rate. Of the patients who received at least BCG induction, 39.7% were tumor-free when those who had early termination of BCG treatment but experienced no recurrence were considered. On the other hand, a substantial portion of the patients (17.3%) experienced BCG intolerance, which could have affected success rates. As expected, planning an RC (or an early RC) was the most preferred treatment after BCG failure. Nonetheless, more than one-fourth of the patients scheduled for this procedure could not undergo surgery due to unfitness or a refusal of this complex operation.

Interestingly, the use of adjuvant treatment for intermediate-risk patients was observed to be suboptimal. Of 1063 intermediate-risk patients, only 2.1% received adequate BCG induction plus maintenance. This low rate can be attributed to the less frequent preference of urologists to give BCG to this group, which does not experience HG tumors according to the previous risk stratification of the EAU Guidelines, and the recent BCG shortage. Intravesical chemotherapy was given to only 23% of the patients, with 21.3% receiving adequate induction therapy. Here again, we observed that some centers administered five instillations, as they counted the postoperative SIVIC as the first dose of the induction scheme, while the others gave six. Remarkably, only 1.3% of all indicated patients completed a 1-year maintenance scheme. As intermediate-risk NMIBC consists of tumors with varying characteristics and outcomes, making it a very heterogeneous group, these rates emphasize the need for further studies to define which intermediate-risk patients would benefit most from adjuvant BCG or chemotherapy treatment.

Notably, this study revealed that intermediate-risk NMIBC patients exhibited the poorest RFS outcomes. However, for other survival outcomes, this group fell between the low-risk and high-risk subgroups, as anticipated. The use of the 2019 EAU Guidelines for risk stratification may have influenced this result. Importantly, these findings highlight the well-established heterogeneity of intermediate-risk tumors and underscore the limitations of previous EAU classifications in robustly defining subgroups. This also suggests that incorporating additional factors, as proposed in the International Bladder Cancer Group’s scoring system and substratification model, could enhance patient stratification and guide more tailored adjuvant treatment decisions [28].

Of the 2237 patients in the registry, 14.4% were diagnosed with MIBC at the initial diagnosis, while 117 (5.2%) developed secondary MIBC. The rate of patients with primary MIBC is slightly lower than reported in the literature. However, the rate of 46% for receiving NAC out of 217 RC patients was explicitly different from the literature and the COBLAnCE cohort. This higher NAC rate could be attributed to several factors. Apart from participating in a QCI program and the inherent Hawthorne effect, the treatment of these patients by urologists subspecialized in onco-urology in a multidisciplinary setting likely contributed to the more frequent use of NAC.

Our cohort approach is, of course, not devoid of limitations. This registry requires a specific electronic file system (KWS), or the software must be adapted to integrate with other hospital electronic systems, necessitating additional financial, labor, and time investments. Currently, only one center is prospectively collecting bodily materials (urine, blood, and fresh-frozen tumor tissue). However, it is important to note that the primary goal of this project is not biosample collection. This aspect has recently started at one center, with plans to expand the biosample collection to other centers. The lack of a central review of pathology reports can be counted as another limitation. However, as this project’s main aim is to report on QCIs and real-world data, we believe a central review is not essential. Moreover, such a review is impractical, as this project is intended to continue indefinitely. Nevertheless, tumor specimens are typically evaluated by pathologists experienced in uropathology, and most cases are discussed at MDTs, which we believe mitigates this limitation. Last, as this registry was created specifically for QCIs, the inherent Hawthorne effect might have affected the results.

## 5. Conclusions

The cohort of the VZNKUL–NMIBC Quality Indicators Program serves as an invaluable source not only for assessing adherence to QCIs but also for evaluating treatment patterns and outcomes in a real-world data setting. This cohort and QCI program are instrumental in increasing the quality of the treatment NMIBC patients receive, improving our understanding of the course of NMIBC under guideline-recommended, state-of-the-art care, and addressing unmet needs in NMIBC management. By reporting on this QCI registry cohort, we aim to foster collaboration with other international projects for data and biosample sharing and to facilitate performing (multidisciplinary) scientific studies.

## Figures and Tables

**Table 1:** Patient characteristics.

		Total *n* = 2237	
**Gender**	Male	1792	80.1%
	Female	445	19.9%
**Age**	Median (IQR)	73	73–74
	>70 years	1375	61.5%
	≤70 years	862	38.5%
**ASA score**	1	138	6.2%
	2	971	43.4%
	3	592	26.5%
	4	27	1.2%
	Not known	509	22.7%
**CCI**	0	640	33%
	1	507	22.7%
	2	416	18.6%
	3	257	11.5%
	4	122	5.5%
	5	49	2.2%
	≥6	67	3%
	Not known	79	3.5%
**Smoking status**	Never smoked	471	21%
	Former smoker	1066	47.7%
	Active smoker	474	21.2%
	Not known	226	10.1%
**History of pelvic radiotherapy**	No	2106	94.2%
	Yes	128	5.7%
	Not known	3	0.1%
**History of UTUC**		125	5.6%
**History of prior BCG**		155	6.9%
**History of prior chemotherapy**		158	7.1%

All values are given as *n* (%), otherwise mentioned. ASA: American Society of Anesthesiologists; BCG: bacillus Calmette–Guérin; CCI: Charlson comorbidity index; IQR: interquartile range; UTUC: upper tract urothelial carcinoma.

**Table 2:** Peri-operative characteristics.

		Total *n* = 4744	
**Time from Dx to TURBT** (days) median (IQR) (min–max)		19 (10–37) (0–375)	
**Operation duration** (minutes) median (IQR) (min–max)		20 (15–30) (1–301)	
**Use of visual enhancement**	No	4231	89.2%
	Yes, Hexvix	469	9.9%
	Yes, NBI	14	0.3%
	Unknown	30	0.6%
**Macroscopical appearance of tumor**	Superficial	3976	83.8%
	Invasive	619	13%
	NK/NR	149	3.2%
**Macroscopically complete resection ^a^**	No	218	7.4%
	Yes	2674	91%
	NK/NR/NA	47	1.6%
**reTURBT planned ^b^**	No	619	54%
	Yes	527	46%
**reTURBT performed ^c^**	No	46	8.7%
	Yes	481	91.2%
**Bladder perforation**		358	7.5%
**Bleeding**		113	2.4%
**Postoperative SIVIC_requested ^d^**	No	499	32.6%
	Yes	956	62.4%
	NK/NR	78	5%
**Postoperative SIVIC_ordered ^d^**	No	39	2.5%
	Yes	917	59.8%
**Postoperative SIVIC_received ^d^**	No	45	2.9%
	Yes	872	56.9%
**Time to SIVIC ^e^** (minutes) median (IQR) (min–max)		282 (194–467.5) (20–1768)	
**Time from TURBT to PR ^f^** (days) median (IQR) (min–max)		5 (3–7) (1–64)	
**Cases discussed in MDT ^g^**		2875	86.9%
**Time from TURBT to MDT ^h^** (days) median (IQR) (min–max)		12 (7–19) (1–139)	
**Time from PR to MDT ^i^** (days) median (IQR) (min–max)		7 (3–12) (0–134)	
**Follow-up time** (months) median (IQR) (min–max)		57 (35–83) (0–136)	

All values are given as *n* (%), otherwise mentioned. Dx: diagnosis; IQR: interquartile range; max: maximum; MDT: multidisciplinary team meeting; min: minimum; NA: not applicable; NBI: narrow-band imaging; NK: not known; NR: not reported; PR: pathology report; reTURBT: repeat TURBT; SIVIC: single intravesical instillation of chemotherapy; TURBT: transurethral resection of the bladder tumor. ^a^ Total *n* = 2939 (TURBTs with T0, Tx, Tis, ≥T2, and inconclusive result and reTURBTs for ad random biopsy, scar tissue, and no detrusor muscle in previous TURBT excluded). ^b^ Total *n* = 1146 (TURBTs with incomplete resection, macroscopically invasive tumor, no urothelial carcinoma, history of high-risk tumor/BCG instillation and reTURBTs for ad random biopsy, scar tissue, and no detrusor muscle in previous TURBT excluded). ^c^ Total *n* = 527 (TURBTs with a planned reTURBT, out of 1146 TURBTs with a reTURBT indication). ^d^ Total *n* = 1533 (TURBTs with incomplete resection, macroscopically invasive appearance, suspicion of no tumor, history of previous high-grade tumor and/or BCG instillation and reTURBTs for ad random biopsy, scar tissue, and no detrusor muscle in previous TURBT excluded). ^e^ Total *n* = 673 (for the hospitals with a value of NA, data were available for only fewer than five patients per center; therefore, not calculated). ^f^ Total *n* = 4259 (TURBTs without available data excluded). ^g^ Total *n* = 3011 (TURBTs with T0, Tx, Tis, inconclusive result, and no PR excluded). ^h^ Total *n* = 2872 (TURBTs without available data excluded). ^i^ Total *n* = 2839 (TURBTs without available data excluded).

**Table 3:** Reasons for not requesting postoperative SIVIC.

Reasons	*n* = 499	
Surgeon’s choice	153	30.7%
Bladder perforation (overt/suspicious)	95	19%
Deep/extensive resection	65	13%
Need for continuous irrigation	45	9%
Patient comorbidity	18	3.6%
Deep resection + continuous irrigation (+/− perforation)	17	3.4%
Concomitant TURP	7	1.4%
Already received/receiving MMC at the moment	7	1.4%
Patient age	6	1.2%
Other	12	2.4%
Not known	74	14.8%

All values are given as *n* (%). MMC: Mitomycin C; SIVIC: single intravesical instillation of chemotherapy; TURP: transurethral resection of the prostate.

**Table 4:** Clinical and histopathological tumor characteristics.

		Total *n* = 4744	
**Tumor size**	≤1 cm	1265	26.6%
	1–3 cm	1280	27%
	≥3 cm	985	20.8%
	NA/NK	1214	25.6%
**Tumor number** median (IQR) (min–max)		2 (1–3) (1–50)	
**Tumor multiplicity**	Single	1760	37.1%
	2–7	2076	43.8%
	≥8	266	5.6%
	NA/NK	642	13.5%
**Tumor localization ^a^**	Base ^b^	976	13.4%
	Posterior wall	1201	16.5%
	Dome	899	12.3%
	Anterior wall	331	4.5%
	Left wall	1447	19.9%
	Right wall	1448	19.9%
	Bladder neck	749	10.3%
	Prostatic loge	234	3.2%
**Total tumor number per localization ^a^**	Base ^b^	1514	13%
	Posterior wall	2030	17.4%
	Dome	1696	14.5%
	Anterior wall	595	5.1%
	Left wall	2112	18.1%
	Right wall	2283	19.5%
	Bladder neck	1107	9.5%
	Prostatic loge	342	2.9%
**Tumor shape**	NK/NR/NA	887	18.7%
	Pws	128	2.7%
	Pbb/N/S	3283	69.2%
	Flat	446	9.4%
**Tumor in diverticulum**	No	4035	85.1%
	Yes	78	1.6%
	NA	631	13.3%
**Tumor stage**	T0	976	20.6%
	Tis	293	6.2%
	Ta	1891	39.8%
	T1	682	14.4%
	≥T2	439	9.3%
	Tx	6	0.1%
	Inconclusive ^c^	30	0.6%
	No PR	427	9%
**Tumor grade (WHO1973)**	Grade 1	334	7%
	Grade 2	445	9.4%
	Grade 3	588	12.4%
	No tumor	977	20.6%
	Not reported	1973	41.6%
	No PR	427	9%
**Tumor grade (WHO2004/2016)**	PUNLMP	232	4.9%
	LG	1042	21.9%
	HG	1289	27.2%
	No tumor	977	20.6%
	Not reported	777	16.4%
	No PR	427	9%
**PRs with both grading systems ^d^**		828	25%
**Tumor-grade reporting ^e^**	TaGx	11	0.6%
	TaPUNLMP	231	12.2%
	TaLG	1010	53.4%
	TaHG	506	26.8%
	TaG1 ^f^	47	2.5%
	TaG2 ^f^	70	3.7%
	TaG3 ^f^	16	0.8%
	T1Gx	17	2.5%
	T1PUNLMP	1	0.2%
	T1LG	22	3.2%
	T1HG	494	72.4%
	T1G1 ^f^	1	0.2%
	T1G2 ^f^	35	5.1%
	T1G3 ^f^	112	16.4%
**Risk stratification (at TURBT level) ^g^**	Low-risk	259	7.9%
	Intermediate-risk	1451	44%
	High-risk	1030	31.3%
	Highest-risk	554	16.8%
**Risk stratification (at patient level) ^h^**	Low-risk	259	14.3%
	Intermediate-risk	670	36.9%
	High-risk	520	28.6%
	Highest-risk	368	20.3%
**Concomitant CIS present** **^i^**		309	10.3%
**Detrusor muscle in specimen ^j^**	Not present	338	21.1%
	Present	1069	66.9%
	NR	192	12%
**LVI ^k^**	Not present	512	15.5%
	Present	93	2.8%
	NR	2700	81.7%
**Variant histology present ^l^**		160	9.7%

All values are given as *n* (%), otherwise mentioned. CIS: carcinoma in situ; IQR: interquartile range; G: grade; HG: high grade; LG: low grade; LVI: lymphovascular invasion; max: maximum; min: minimum; N: nodular tumor; NA: not applicable; NK: not known; NR: not reported; Pbb: papillary tumor, broad-based; PR: pathology report; PUNLMP: papillary urothelial neoplasm of low malignant potential; Pws: papillary tumor with stalk; S: sessile tumor; WHO: World Health Organization. ^a^ Total *n* = 4017 (reTURBTs for ad random biopsy, scar tissue, incomplete previous TURBT, and no detrusor muscle in previous TURBT excluded). ^b^ Base includes trigon and ureter orifices. ^c^ Inconclusive includes biopsy/coagulation artifact and no viable tissue/mucosa. ^d^ Total *n* = 3311 (TURBTs with T0, inconclusive result, and no PR excluded). ^e^ Total *n* = 2866 (TURBTs with T0, Tx, ≥T2, inconclusive result, and no PR excluded). ^f^ Only the WHO1973 grading system was used (for the others, both grading systems were used). ^g^ Total *n* = 3294 (TURBTs with T0, Tx, ≥T2, and inconclusive result excluded). ^h^ Total *n* = 1817 (according to the first registered TURBT, patients with T0, Tx, ≥T2, and inconclusive result excluded). ^i^ Total *n* = 3011 (TURBTs with T0, Tx, Tis, inconclusive result, and no PR excluded). ^j^ Total *n* = 1599 (TURBTs with T0, Tx, Tis, TaLG/G1-2, inconclusive result, and no PR and reTURBTs for ad random biopsy and scar tissue excluded). ^k^ Total *n* = 3305 (TURBTs with T0, Tx, inconclusive result, and no PR excluded). ^l^ Total *n* = 1643 (TURBTs with T0, Tx, Tis, TaLG/G1-2, inconclusive result, and no PR excluded).

**Table 5:** Adjuvant intravesical treatment status of BCG-indicated tumors.

**Treatment**	**T1 Tumors** ***n* = 478**	**TaHG Tumors** ***n* = 376**	**Tis Tumors** ***n* = 118**	**All HR Tumors** ***n* = 972**	**All IR Tumors** ***n* = 1063**	
BCG induction planned	388 (81.2%)	276 (73.4%)	104 (88.2%)	768 (79%)	41 (3.9%)	
BCG induction received	327 (68.4%)	201 (53.5%)	96 (81.4%)	624 (64.2%)	35 (3.3%)	
Adequate BCG induction	311 (65.1%)	189 (50.3%)	90 (76.3%)	590 (60.7%)	33 (3.1%)	
BCG maintenance planned	246 (51.5%)	131 (34.8%)	62 (52.5%)	439 (45.2%)	22 (2.1%)	
BCG maintenance received	217 (45.4%)	116 (30.9%)	57 (48.3%)	390 (40.1%)	22 (2.1%)	
Adequate BCG maintenance	214 (44.8%)	114 (30.3%)	55 (46.6%)	383 (39.4%)	22 (2.1%)	
BCG maintenance <1-year	83 (17.4%)	40 (10.6%)	22 (18.6%)	146 (15%)	17 (1.6%)	
BCG maintenance 1-year	75 (15.7%)	46 (12.2%)	24 (20.3%)	145 (14.9%)	3 (0.3%)	
BCG maintenance 2-year ^a^	38 (8%)	20 (5.3%)	5 (4.2%)	63 (6.5%)	1 (0.1%)	
BCG maintenance 3-year ^b^	17 (3.6%)	8 (2.1%)	4 (3.4%)	29 (3%)	1 (0.1%)	
Chemotherapy received/1-year ^c^	23 (4.8%)	44 (11.7%)	5 (4.2%)	72 (7.4%)	245 (23%)	14 (1.3%)

All values are given as *n* (%). ^a^ 2-year: maintenance more than 1 year but less than or equal to 2 years. ^b^ 3-year: maintenance more than 2 year but less than or equal to 3 years. ^c^ Only for intermediate-risk tumors. BCG: bacillus Calmette-Guérin, HG: high grade, HR: high-risk, IR: intermediate-risk.

**Table 6:** Management of high-risk tumors and results of intravesical BCG treatments.

**T1 (*n* = 478)**	**BCG Result**	**Adequate BCG** **214 (44.8%)**		**No maint.** **113 (23.6%)**		**BCG induction not planned/not received** **151 (31.6%)**							
	Success	95	(19.8%)	24	(5%)			**AIC (23; 4.9%)**		**NT (114; 23.8%)**		**eRC (14; 2.9%)**	
	Intolerance w/o recurrence	58	(12.1%)	16	(3.4%)	No recurrence		9	(2%)	56	(11.8%)	11	(2.3%)
	Intolerance w/recurrence	10	(2.1%)	9	(1.9%)	Recurrence	TaLG	1	(0.2%)	13	(2.8%)	-	-
	Refractory	25	(5.2%)	37	(7.7%)		TaHG	1	(0.2%)	8	(1.6%)	-	-
	Relapsing	5	(1.1%)	-	-		Tis	2	(0.4%)	8	(1.6%)	-	-
	MIBC	8	(1.7%)	22	(4.6%)		T1	5	(1.1%)	8	(1.6%)	-	-
	Metastasis	5	(1.1%)	2	(0.4%)	MIBC		4	(0.8%)	13	(2.8%)	-	-
	Patient died ^a^	8	(1.7%)	3	(0.6%)	Metastasis		1	(0.2%)	8	(1.6%)	3	(0.6%)
**TaHG (*n* = 376)**	**BCG result**	**Adequate BCG** **114 (30.3%)**		**No maint.** **87 (23.1%)**		**BCG induction not planned/not received** **175 (46.5%)**							
	Success	55	(14.6%)	41	(10.9%)			**AIC (43; 11.5%)**		**NT (130; 34.6%)**		**eRC (2; 0.5%)**	
	Intolerance w/o recurrence	33	(8.8%)	8	(2.1%)	No recurrence		17	(4.5%)	43	(11.4%)	2	(0.5%)
	Intolerance w/recurrence	6	(1.6%)	3	(0.8%)	Recurrence	TaLG	7	(1.9%)	28	(7.5%)	-	-
	Refractory	7	(1.9%)	25	(6.7%)		TaHG	3	(0.8%)	33	(8.8%)	-	-
	Relapsing	7	(1.9%)	6	(1.6%)		Tis	10	(2.7%)	6	(1.6%)	-	-
	MIBC	3	(0.8%)	2	(0.5%)		T1	3	(0.8%)	8	(2.1%)	-	-
	Metastasis	-	-	1	(0.3%)	MIBC		3	(0.8%)	10	(2.7%)	-	-
	Patient died ^a^	3	(0.8%)	1	(0.3%)	Metastasis		-	-	2	(0.5%)	-	-
**Tis (*n* = 118)**	**BCG result**	**Adequate BCG** **55 (46.6%)**		**No maint.** **40 (33.9%)**		**BCG induction not planned/not received** **22 (18.6%)**							
	Success	24	(20.3%)	14	(11.9%)			**AIC (5; 4.2%)**		**NT (10; 8.4%)**		**eRC (7; 5.9%)**	
	Intolerance w/o recurrence	17	(14.4%)	1	(0.8%)	No recurrence		1	(0.8%)	4	(3.4%)	7	(5.9%)
	Intolerance w/recurrence	2	(1.7%)	2	(1.7%)	Recurrence	TaLG	1	(0.8%)	1	(0.8%)	-	-
	Refractory	4	(3.4%)	14	(11.9%)		TaHG	-	-	-	-	-	-
	Relapsing	4	(3.4%)	6	(5.1%)		Tis	1	(0.9%)	3	(2.5%)	-	-
	MIBC	2	(1.7%)	2	(1.7%)		T1	2	(1.7%)	-	-	-	-
	Metastasis	-	-	1	(0.8%)	MIBC		-	-	2	(1.7%)	-	-
	Patient died ^a^	2	(1.7%)	-	-	Metastasis		-	-	-	-	-	-
	**BCG result**	**Adequate BCG** **383 (39.4%)**		**No maint.** **240 (24.7%)**		**BCG induction not planned/not received** **348 (35.8%)**							
**All HR tumors (*n* = 972)**	Success	174	(17.9%)	79	(8.1%)			**AIC (71; 7.3%)**		**NT (254; 26.1%)**		**eRC (23; 2.4%)**	
	Intolerance w/o recurrence	108	(11.1%)	25	(2.6%)	No recurrence		27	(2.8%)	103	(10.6%)	20	(2.1%)
	Intolerance w/recurrence	18	(1.9%)	14	(1.4%)	Recurrence	TaLG	9	(0.9%)	42	(4.3%)	-	-
	Refractory	36	(3.7%)	76	(7.8%)		TaHG	4	(0.4%)	41	(4.2%)	-	-
	Relapsing	16	(1.7%)	12	(1.2%)		Tis	13	(1.4%)	17	(1.8%)	-	-
	MIBC	13	(1.3%)	26	(2.7%)		T1	10	(1%)	16	(1.6%)	-	-
	Metastasis	5	(0.5%)	4	(0.4%)	MIBC		7	(0.7%)	25	(2.6%)	-	-
	Patient died ^a^	13	(1.3%)	4	(0.4%)	Metastasis		1	(0.1%)	10	(1%)	3	(0.3%)

All values are given as *n* (%). Percentages are calculated according to the number of all BCG-indicated patients in each group and in total. Some percentages are rounded. ^a^ Patient died during the treatment. AIC: adjuvant intravesical chemotherapy, BCG: bacillus Calmette-Guérin, eRC: early radical cystectomy, HG: high grade, HR: high-risk, LG: low grade, MIBC: muscle-invasive bladder cancer, NT: not treated, w/: with, w/o: without.

**Table 7:** Further first-line management of patients with BCG failure.

Disease Group	BCG Failure Type	* n *	No Active Treatment	BCG RechalLenge	BCG Continuation	Intravesical Chemo	(Early) Cx Proposed
**T1** ***n*=116**	Intolerance w/rec.	19 (15.3%)	3 (15.8%)	4 (21.1%)	1 (5.2%)	3 (15.8%)	8 (42.1%)
	Refractory	62 (53.4%)	12 (19.4%)	18 (29%)	3 (4.8%)	-	29 (46.8%)
	Relapsing	5 (4.2%)	1 (20%)	2 (40%)	-	-	2 (40%)
	MIBC	30 (27.1%)	5 (16.7%)	-	-	-	25 (83.3%)
**TaHG** ***n*=59**	Intolerance w/rec.	9 (14.3%)	5 (55.6%)	1 (11.1%)	-	-	3 (33.3%)
	Refractory	32 (57.1%)	7 (21.8%)	12 (37.5%)	2 (6.3%)	-	11 (34.4%)
	Relapsing	13 (20.7%)	9 (69.2%)	1 (7.7%)	-	-	3 (23.1%)
	MIBC	5 (7.9%)	2 (40%)	-	-	-	3 (60%)
**Tis** ***n*=36**	Intolerance w/rec.	4 (10.3%)	1 (25%)	3 (75%)	-	-	-
	Refractory	18 (43.6%)	1 (5.9%)	10 (58.9%)	-	1 (5.9%)	6 (29.4%)
	Relapsing	10 (35.9%)	3 (30%)	5 (50%)	-	-	2 (20%)
	MIBC	4 (10.3%)	1 (25%)	-	-	-	3 (75%)
**All tumors** ***n*=211**	Intolerance w/rec.	32 (14.1%)	9 (28.1%)	8 (25%)	1 (3.1%)	3 (9.4%)	11 (34.4%)
	Refractory	112 (52.7%)	20 (17.8%)	40 (35.7%)	5 (4.5%)	1 (0.9%)	46 (41.1%)
	Relapsing	28 (14.6%)	13 (46.3%)	8 (28.6%)	-	-	7 (25%)
	MIBC	39 (18.6%)	8 (20.5%)	-	-	-	31 (79.5%)
	**Total**	**211 (100%)**	**50 (23.7%)**	**56 (26.5%)**	**6 (2.9%)**	**4 (1.9%)**	**99 (45%)**

All values are given as *n* (%). The percentages for management options refer to the total number (*n*) of that row, while the percentages of *n* refer to the total number of patients in the subgroup. BCG: bacillus Calmette-Guérin, Cx: cystectomy, HG: high grade, MIBC: muscle-invasive bladder cancer, w/rec.: with recurrence.

**Table 8:** Tumor characteristics and treatment of patients with MIBC.

		*n* (%)	
Non-metastatic primary MIBC ^a^		283	12.7%
Non-metastatic secondary MIBC ^a^		117	5.2%
de novo metastatic UC ^a^		39	1.7%
Upstaging from T1 disease to MIBC ^b^		21	8.4%
Undergoing RC ^c^		217	54.3%
Receiving NAC ^d^		100	46%
Tumor stage	Tx	4	1.8%
	T0	46	21.2%
	Tis	12	5.5%
	Ta	2	0.9%
	T1	8	3.7%
	T2a	18	8.3%
	T2b	22	10.1%
	T3a	39	18%
	T3b	40	18.4%
	T4a	22	10.1%
	T4b	4	1.8%
Node stage	Nx	9	4.1%
	N0	157	72.4%
	N1	19	8.8%
	N2	27	12.4%
	N3	5	2.3%
Variant histology		75	17%
Bi-/trimodality treatment ^c^		30	7.5%
Palliative RT ^c^		21	5.3%
Palliative treatment/BSC/FU ^c^		123	30.8%
Unknown ^c^		9	2.3%

All values are given as *n* (%), otherwise mentioned. BSC: best supportive care; FU: follow-up; MIBC: muscle-invasive bladder cancer; NAC: neoadjuvant chemotherapy; RC: radical cystectomy; RT: radiotherapy; UC: urothelial cancer. ^a^ Total *n* = 2237 (unique patient number). ^b^ Total *n* = 251 (T1 patients underwent reTURBT). ^c^ Total *n* = 400 (non-metastatic MIBC patients). ^d^ Total *n* = 217 (patients underwent RC).

**Table 9:** Two-, five-, and ten-year survival estimates for all NMIBC patients and subgroups.

**2-Year**	**RFS**	**PFS**	**CFS**	**OS**	**CSS**
**All tumors**	**63.4** **(61–65.9)**	**94.9** **(93.7–95.8)**	**92.3** **(91–93.5)**	**88.2** **(86.6–89.6)**	**98.3** **(97.6–98.8)**
Low-risk	80.3 (75.5–85.5)	99.6 (97.1–99.9)	100	91.8 (87.8–94.6)	100
Intermediate-risk	68.6 (65–72.3)	98.4 (97.1–99.2)	99.2 (98.1–99.7)	90.5 (88–92.5)	99.6 (98.8–99.9)
High-risk	74.3 (70.5–78.4)	95.3 (93–96.8)	91.8 (89–93.9)	90.1 (87.2–92.4)	98.3 (96.8–99.1)
Highest-risk	80.8 (76.5–85.3)	84 (79.6–87.6)	76.4 (71.4–80.6)	78.6 (74–82.4)	94.8 (92.1–96.7)
**5-year**	**RFS**	**PFS**	**CFS**	**OS**	**CSS**
**All tumors**	**53** **(50.6–55.5)**	**91.6** **(90–92.9)**	**89** **(87.3–90.4)**	**70.6** **(68.4–72.7)**	**95.6** **(94.5–96.5)**
Low-risk	66 (60–72.5)	99 (95.9–99.8)	99.4 (95.7–99.9)	80.6 (75.2–85)	100
Int-risk	51.9 (47.9–56.3)	96.1 (94–97.5)	97.3 (95.4–98.4)	74.1 (70.5–77.4)	98.5 (97.3–99.3)
High-risk	63.8 (59.3–68.7)	90.7 (87.6–93)	86.4 (82.7–89.3)	68.7 (64.3–72.7)	94.9 (92.7–96.6)
Highest-risk	80.1 (75.7–84.8)	78.6 (73.5–82.8)	72.3 (67–77)	59.6 (54.2–64.5)	87.9 (84.3 – 91.1)
**10-year**	**RFS**	**PFS**	**CFS**	**OS**	**CSS**
**All tumors**	**47.3** **(44–50.8)**	**87.6** **(84.7–89.9)**	**85.8** **(82.2–88.7)**	**45.1** **(41–49.2)**	**92.9** **(91–94.5)**
Low-risk	56 (45–69.6)	92.1 (74.3–97.7)	98.7 (94.9–99.7)	57.2 (45.2–67.4)	99.3 (96.4–99.9)
Int-risk	42.6 (36–50.4)	93.9 (91–95.9)	84.9 (72.3–92.1)	48.2 (40.1–55.3)	97.5 (95.8–98.6)
High-risk	62 (57.2–67.2)	87 (82.5–90.4)	83.6 (78.9–87.4)	43.6 (36.1–50.9)	89.8 (84.8–93.7)
Highest-risk	76 (69–83.8)	73.2 (65.9–79.1)	67.3 (60.8–73)	32.9 (25–41.1)	84.4 (79.7–88.6)

All values are given as percentages (%) and 95% confidence intervals. CFS: cystectomy-free survival; CSS: cancer-specific survival; NMIBC: non-muscle-invasive bladder cancer; OS: overall survival; PFS: progression-free survival; RFS: recurrence-free survival.

## Data Availability

Data are available upon reasonable request. Researchers may request access to data by contacting the corresponding author.

## Supplementary Materials

The following supporting information can be downloaded at https://www.mdpi.com/article/10.3390/cancers16213653/s1: Table S1: (a) number of TURBTs per center per year; (b) number of unique patients for TURBTs per center per year. Table S2: (a) number of follow-ups per center per year; (b) number of unique patients for follow-ups per center per year. Table S3: (a) number of multidisciplinary team meetings per center per year; (b) number of unique patients for multidisciplinary team meetings per center per year. Table S4: (a) number of bladder instillations per center per year; (b) Number of unique patients for bladder instillations per center per year. Table S5: (a) distribution of benign conditions; (b) distribution of bladder cancers other than urothelial carcinoma; (c) distribution of cancers other than bladder cancer. Table S6: distribution of the cases with a variant histology type.

## Abbreviations

AJCC	American Joint Committee on Cancer
ASA	American Society of Anesthesiologists
BC	Bladder cancer
BCG	Bacillus Calmette–Guérin
CCI	Charlson comorbidity index
CFS	Cystectomy-free survival
CIS	Carcinoma in situ
COBLAnCE	A Cohort to Study Bladder Cancer
CR	Complete resection
CSS	Cancer-specific survival
DM	Detrusor muscle
EAU	European Association of Urology
eCRF	Electronic case report form
FDA	Food and Drug Administration
HG	High grade
IQR	Interquartile range
KWS	Klinisch Werkstation
LG	Low grade
LVI	Lymphovascular invasion
MDT	Multidisciplinary team
MIBC	Muscle-invasive bladder cancer
NAC	Neoadjuvant chemotherapy
NMIBC	Non-muscle-invasive bladder cancer
OS	Overall survival
PFS	Progression-free survival
QCI	Quality control indicator
RC	Radical cystectomy
RESECT	Transurethral REsection and Single-instillation intra-vesical chemotherapy Evaluation in bladder Cancer Treatment
reTURBT	Repeat TURBT
RFS	Recurrence-free survival
SIVIC	Single intravesical instillation of chemotherapy
TNM	Tumor-node-metastasis
TURBT	Transurethral resection of the bladder tumor
UC	Urothelial carcinoma
UTUC	Upper tract urothelial carcinoma
VZNKUL	Vlaams Ziekenhuisnetwerk–KU Leuven
WHO	World Health Organization
