# Supplementary material for: Cohort Profile: VZNKUL–NMIBC Quality Indicators Program: A Flemish Prospective Cohort to Evaluate the Quality Indicators in the Treatment of Non-Muscle-Invasive Bladder Cancer
Source: Cancers (Basel). 2024 Oct 29;16(21):3653. doi: 10.3390/cancers16213653 (PMC11545168; doi:10.3390/cancers16213653)
Supplement: Supplementary file 1 [file cancers-16-03653-s001.zip › Supp.Table S1.pdf]

**Supplementary Table S1-a:** Number of TURBTs per center per year.

| Years | HOSP-1 | HOSP-2 | HOSP-3 | HOSP-4 | HOSP-5 | HOSP-6 | HOSP-7 | Total |
|---|---|---|---|---|---|---|---|---|
| **2013** | 76 | 35 | 10 | | | | | 121 |
| **2014** | 166 | 76 | 12 | 42 | | | | 296 |
| **2015** | 178 | 100 | 8 | 97 | | | | 383 |
| **2016** | 251 | 135 | | 93 | | | | 479 |
| **2017** | 268 | 148 | | 109 | 32 | | | 557 |
| **2018** | 314 | 140 | 3 | 125 | 34 | 61 | | 677 |
| **2019** | 306 | 151 | | 142 | 62 | 40 | 77 | 778 |
| **2020** | 284 | 170 | | 131 | 74 | 22 | 53 | 734 |
| **2021** | 291 | 185 | | 158 | 62 | 5 | 95 | 796 |
| **2022** | 282 | 221 | | 116 | 59 | 63 | 79 | 820 |
| **2023** | 241 | 219 | | 122 | 56 | 15 | 95 | 748 |
| **2024** | 106 | 80 | | 32 | 19 | | 21 | 260 |
| **Total** | **2763** | **1660** | **33** | **1167** | **398** | **206** | **420** | **6666** |

**Supplementary Table S1-b:** Number of unique patients for TURBTs per center per year.

| Years | HOSP-1 | HOSP-2 | HOSP-3 | HOSP-4 | HOSP-5 | HOSP-6 | HOSP-7 | Total |
|---|---|---|---|---|---|---|---|---|
| **2013** | 72 | 35 | 9 | | | | | 116 |
| **2014** | 150 | 72 | 10 | 42 | | | | 274 |
| **2015** | 157 | 96 | 8 | 88 | | | | 349 |
| **2016** | 214 | 129 | | 84 | | | | 427 |
| **2017** | 211 | 130 | | 97 | 28 | | | 466 |
| **2018** | 242 | 124 | 3 | 109 | 30 | 56 | | 564 |
| **2019** | 234 | 133 | | 123 | 54 | 38 | 71 | 652 |
| **2020** | 238 | 147 | | 115 | 67 | 21 | 46 | 635 |
| **2021** | 226 | 161 | | 130 | 53 | 5 | 81 | 665 |
| **2022** | 218 | 191 | | 104 | 52 | 57 | 71 | 694 |
| **2023** | 194 | 174 | | 111 | 51 | 15 | 87 | 635 |
| **2024** | 100 | 76 | | 30 | 19 | | 21 | 247 |
| **Total** | **1502** | **1129** | **27** | **682** | **254** | **176** | **324** | **4104** |
