# Supplementary material for: Cohort Profile: VZNKUL–NMIBC Quality Indicators Program: A Flemish Prospective Cohort to Evaluate the Quality Indicators in the Treatment of Non-Muscle-Invasive Bladder Cancer
Source: Cancers (Basel). 2024 Oct 29;16(21):3653. doi: 10.3390/cancers16213653 (PMC11545168; doi:10.3390/cancers16213653)
Supplement: Supplementary file 1 [file cancers-16-03653-s001.zip › Supp.Table S2.pdf]

Supplementary Table S2-a: Number of follow-ups per center per year.

Years	HOSP-1	HOSP-2	HOSP-3	HOSP-4	HOSP-5	HOSP-6	HOSP-7	Total
**2013**	6						1	7
**2014**	12	20	2				1	35
**2015**	12	139	9					160
**2016**	12	145	9					166
**2017**	10	164	9	5				188
**2018**	15	162	6	2				185
**2019**	170	187	1					358
**2020**	283	141	3					427
**2021**	135	89	1		1			226
**2022**	120	203	3		2			328
**2023**	173	206	8					387
**2024**	17	65	3					87
**Total**	**965**	**1506**	**54**	**7**	**3**		**2**	**2554**

Supplementary Table S2-b: Number of unique patients for follow-ups per center per year.

Years	HOSP-1	HOSP-2	HOSP-3	HOSP-4	HOSP-5	HOSP-6	HOSP-7	Total
**2013**	5						1	6
**2014**	9	19	2				1	31
**2015**	10	92	6					108
**2016**	10	91	6					107
**2017**	9	103	7	4				123
**2018**	12	103	5	1				120
**2019**	144	135	1					279
**2020**	206	113	3					321
**2021**	118	83	1		1			203
**2022**	106	175	3		2			286
**2023**	134	174	3					311
**2024**	17	64	3					86
**Total**	**453**	**646**	**25**	**4**	**3**		**2**	**1136**
