# Supplementary material for: Cohort Profile: VZNKUL–NMIBC Quality Indicators Program: A Flemish Prospective Cohort to Evaluate the Quality Indicators in the Treatment of Non-Muscle-Invasive Bladder Cancer
Source: Cancers (Basel). 2024 Oct 29;16(21):3653. doi: 10.3390/cancers16213653 (PMC11545168; doi:10.3390/cancers16213653)
Supplement: Supplementary file 1 [file cancers-16-03653-s001.zip › Supp.Table S3.pdf]

Supplementary Table S3-a: Number of MDTs per center per year.

Years	HOSP-1	HOSP-2	HOSP-3	HOSP-4	HOSP-5	HOSP-6	HOSP-7	Total
**2016**	7							7
**2017**	122		22	7				151
**2018**	75		58					133
**2019**	204		52	1				257
**2020**	224		42	49				315
**2021**	220		4	55	9			288
**2022**	218		63	49				330
**2023**	207		105	57				369
**2024**	83		27	23				133
**Total**	**1360**		**373**	**241**	**9**			**1983**

Supplementary Table S3-b: Number of unique patients for MDTs per center per year.

Years	HOSP-1	HOSP-2	HOSP-3	HOSP-4	HOSP-5	HOSP-6	HOSP-7	Total
**2016**	7							7
**2017**	108		22	7				137
**2018**	64		54					118
**2019**	162		47	1				210
**2020**	192		42	46				280
**2021**	177		4	47	9			237
**2022**	177		57	44				278
**2023**	165		96	51				312
**2024**	81		26	22				129
**Total**	**830**		**322**	**159**	9			**1293**
