# Supplementary material for: Cohort Profile: VZNKUL–NMIBC Quality Indicators Program: A Flemish Prospective Cohort to Evaluate the Quality Indicators in the Treatment of Non-Muscle-Invasive Bladder Cancer
Source: Cancers (Basel). 2024 Oct 29;16(21):3653. doi: 10.3390/cancers16213653 (PMC11545168; doi:10.3390/cancers16213653)
Supplement: Supplementary file 1 [file cancers-16-03653-s001.zip › Supp.Table S4.pdf]

Supplementary Table S4-a: Number of bladder instillations per center per year.

Years	HOSP-1	HOSP-2	HOSP-3	HOSP-4	HOSP-5	HOSP-6	HOSP-7	Total
**2016**	516							516
**2017**	773			66				839
**2018**	628			162				790
**2019**	711			284				995
**2020**	769	451		381				1601
**2021**	834	891		461	1			2187
**2022**	626	854	74	527	283			2364
**2023**	761	760	314	415	558			2808
**2024**	246	281	70	141	217			955
**Total**	**5864**	**3237**	**458**	**2437**	**1059**			**13055**

Supplementary Table S4-b: Number of unique patients for bladder instillations per center per year.

Years	HOSP-1	HOSP-2	HOSP-3	HOSP-4	HOSP-5	HOSP-6	HOSP-7	Total
**2016**	108							108
**2017**	127			18				145
**2018**	94			32				126
**2019**	112			47				159
**2020**	122	90		64				276
**2021**	113	134		69	1			317
**2022**	93	144	22	69	65			393
**2023**	101	114	61	67	86			429
**2024**	63	73	18	44	62			260
**Total**	**501**	**331**	**81**	**169**	**132**			**1114**
