# Supplementary material for: Cohort Profile: VZNKUL–NMIBC Quality Indicators Program: A Flemish Prospective Cohort to Evaluate the Quality Indicators in the Treatment of Non-Muscle-Invasive Bladder Cancer
Source: Cancers (Basel). 2024 Oct 29;16(21):3653. doi: 10.3390/cancers16213653 (PMC11545168; doi:10.3390/cancers16213653)
Supplement: Supplementary file 1 [file cancers-16-03653-s001.zip › Supp.Table S5.pdf]

Supplementary Table S5-a: Distribution of benign conditions.

Benign conditions	*n*=251	
**Acute cystitis**	2	0.8%
**Acute inflammation**	3	1.2%
**Amyloid**	2	0.8%
**Chronic cystitis**	10	4%
**Chronic inflammation**	38	15.5%
**Congestion**	1	0.4%
**Cystitis cystica**	13	5.3%
**Cystitis cystica & glandularis**	7	2.8%
**Cystitis follicularis**	1	0.4%
**Cystitis glandularis**	7	2.8%
**Dysplasia**	1	0.4%
**Edematous ulcerated tissue**	3	1.2%
**Endometriosis**	3	1.2%
**Eosinophilic cystitis**	6	2.4%
**Epithelial reactive atypia**	1	0.4%
**Erosive cystitis**	3	1.2%
**Fibrous polyp**	1	0.4%
**Fibrous stroma**	2	0.8%
**Granulation tissue**	2	0.8%
**Inflammatory changes**	5	2%
**Inflammatory pseudopolyp**	1	0.4%
**Inflammatory cystitis**	6	2.4%
**Intestinal metaplasia**	1	0.4%
**Inverted papilloma**	6	2.4%
**Leiomyoma**	2	0.8%
**Lipoma**	1	0.4%
**Muscle tissue**	1	0.4%
**Nephrogenic adenoma**	8	3.2%
**Non-specific cystitis**	7	2.8%
**Non-specific cystitis**	4	1.6%
**Normal urothelium**	30	12.6%
**Papillary cystitis**	1	0.4%
**Papillary hyperplasia**	1	0.4%
**Papilloma**	24	9.8%
**Polypoid cystitis**	2	0.8%
**Polypoid urethritis**	1	0.4%
**Prostate tissue**	8	3.2%
**Radiation cystitis**	7	2.8%
**Reactive changes**	17	6.9%
**Schistosomiasis**	1	0.4%
**Squamous metaplasia**	2	0.8%
**Subacute inflammation**	2	0.8%
**Ulcerative mucosa**	2	0.8%
**Urothelial hyperplasia**	4	1.6%
**Villeous adenoma**	1	0.4%

All values are given as *n* (%).

Supplementary Table S5-b: Distribution of bladder cancers other than urothelial carcinoma.

Non-urothelial bladder cancers	*n*=17	
**Spinocellular carcinoma**	8	47.1%
**Neuroendocrine small cell carcinoma**	5	29.4%
**Adenocarcinoma (colloid/mucinous)**	3	17.6%
**Neuroendocrine big cell carcinoma**	1	5.9%

All values are given as *n* (%).

Supplementary Table S5-c: Distribution of cancers other than bladder cancer.

Cancers other than bladder cancer	*n*=36	
**Prostate carcinoma**	20	55.6%
**Colon/sigmoid carcinoma metastasis**	4	11.1%
**Adenocarcinoma (appendix/intestine)**	2	5.6%
**Clear cell renal cell carcinoma metastasis**	2	5.6%
**Cervix carcinoma**	2	5.6%
**Breast carcinoma metastasis**	1	2.8%
**MALT lymphoma**	1	2.8%
**Malign melanoma metastasis**	1	2.8%
**Sarcomatoid carcinoma**	1	2.8%
**Signet cell carcinoma metastasis**	1	2.8%
**Small cell lymphocytic infiltration**	1	2.8%

All values are given as *n* (%). MALT: Mucosa-associated lymphoid tissue.
