# Supplementary material for: Cohort Profile: VZNKUL–NMIBC Quality Indicators Program: A Flemish Prospective Cohort to Evaluate the Quality Indicators in the Treatment of Non-Muscle-Invasive Bladder Cancer
Source: Cancers (Basel). 2024 Oct 29;16(21):3653. doi: 10.3390/cancers16213653 (PMC11545168; doi:10.3390/cancers16213653)
Supplement: Supplementary file 1 [file cancers-16-03653-s001.zip › Supp.Table S6.pdf]

Supplementary Table S6: Distribution of the cases with a variant histology type.

Variant histology types	*n*=70	
**Squamous**	23	32.9%
**Glandular**	20	28.6%
**Micropapillary**	7	10%
**Lymphoepithelioma-like**	4	5.7%
**Micropapillary + glandular**	3	4.3%
**Neuroendocrine/small cell**	2	2.9%
**Spinocellular**	2	2.9%
**Sarcomatoid**	2	2.9%
**Adenocarcinomateous**	1	1.4%
**Nested**	1	1.4%
**Pleomorphic/plasmacytoid**	1	1.4%
**Pseudosarcomatoid**	1	1.4%
**Villoglandular**	1	1.4%
**Villoglandular + micropapillary**	1	1.4%
**Squamous + sarcomatoid**	1	1.4%

All values are given as *n* (%).
